# Cross-Linkable Hydrocarbon Resins Bearing β-Styryl Moieties and Allyl Groups: Synthesis, Curing Behavior and Performance for Copper-Clad Laminate Applications

**DOI:** 10.3390/polym18212672

**Published:** 2026-10-28

**Authors:** Zhaoji Li, Yueqi Mo

**Affiliations:** School of Materials Science and Engineering, South China University of Technology, Guangzhou 510640, China; ms225508691@mail.scut.edu.cn

**Keywords:** hydrocarbon resins, copper-clad laminates (CCLs), low dielectric loss, cross-linkable resin, thermal stability

## Abstract

Currently, hydrocarbon resins are mainly classified into styryl-based and benzocyclobutene (BCB)-based systems. Styryl-based hydrocarbon resins are cost-effective but generally show limited heat resistance, whereas BCB-based resins have relatively high cost. Moreover, these two systems seem to have mismatched curing temperature windows and limited mutual compatibility. Herein, we develop a cross-linkable hydrocarbon resin derivative BP-TVCH from biphenyl and 1,2,4-trivinylcyclohexane, rich in β-styryl moieties and allyl groups. Upon curing, the benzene rings are incorporated into the cross-linked network to improve thermostability. The β-styryl moieties undergo homopolymerization, while the allyl groups participate in curing through chain-transfer reactions. The resulting resin can be co-cured with both styryl-based and BCB-based hydrocarbon resins. When composited with styrene-terminated polyphenylene ether (OPE-2st), the resulting CCL exhibits a dielectric loss of 0.00101–0.00105 at 10 GHz and a high glass transition temperature (Tg) of up to 277 °C.

## 1. Introduction

With post-5G communication technologies continuously evolving toward higher frequency bands and the operating frequencies of AI chips steadily increasing, high-frequency and high-speed copper-clad laminates (CCLs) and IC packaging substrates are imposing increasingly stringent requirements on the dielectric properties of substrate materials. A relatively high dielectric constant (*D*k) or large dielectric loss (*D*f) of the substrate exacerbates signal loss during high-speed transmission and may lead to issues such as device heating. To meet the demands of high-frequency and high-speed circuits, the development of resin matrices with low *D*k and ultra-low *D*f has become an important research direction. Among the available options, the dielectric performance of hydrocarbon resins is second only to that of fluoropolymers such as polytetrafluoroethylene (PTFE), while hydrocarbon resins are significantly superior to fluorinated resins in key aspects, including thermal stability, mechanical properties, and copper foil adhesion. Consequently, hydrocarbon resins have emerged as preferred materials for high-frequency substrate applications.

**Figure 1:**
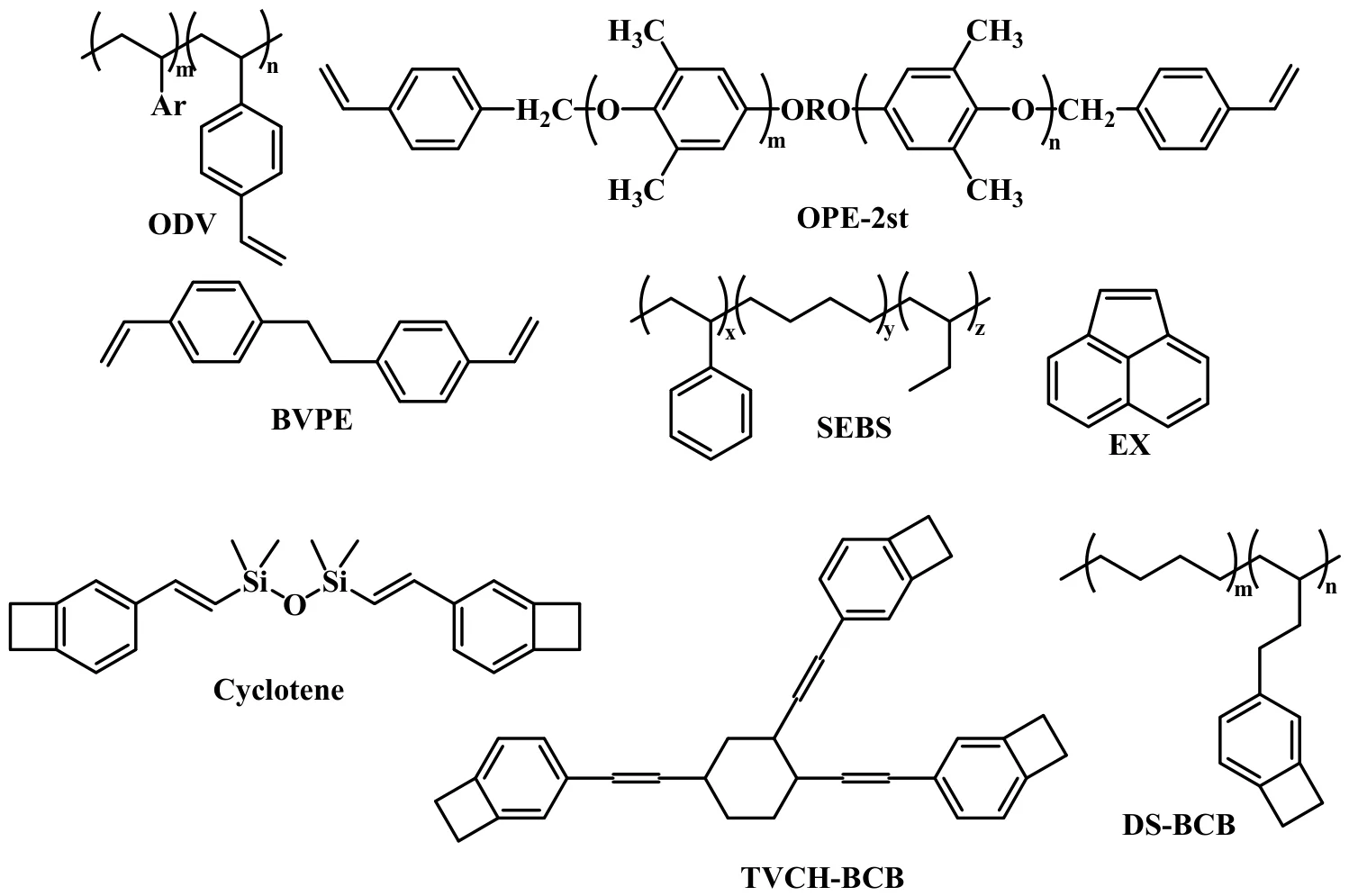
Chemical structures of representative low-*D*k/low-*D*f resins.

The research and application of low-*D*k/low-*D*f resins, as illustrated in Figure 1, can be broadly divided into several stages:

First-generation matrices and modified polyphenylene oxide (PPO). Early hydrocarbon matrices based on polybutadiene, styrene–butadiene–styrene block copolymer (SBS), and hydrogenated styrene–butadiene block copolymer (SEBS) [1,2] exhibit high coefficients of thermal expansion (CTEs) and insufficient heat resistance, often requiring blending with cyclo-olefin copolymers (COCs) [3] or PPO [4,5]. Although PPO is not a pure hydrocarbon resin, its excellent thermal and mechanical properties (*D*f ~ 0.002) and early adoption by General Electric/Benz [6] established it as a key modifying resin. Modified PPOs for low-dielectric CCLs mainly include the early allyl PPO from Asahi Kasei [7], which exhibits good overall performance but requires a high curing temperature, and the currently mainstream styrene-terminated OPE-2st [8], which has a low synthesis cost and a curing window better matched to industrial processes.

Second-generation ODV, EX, and BVPE. Second-generation resins are represented by ODV (styrene–divinylbenzene copolymer) from Nippon Steel Chemical [9,10,11,12], in which DVB (divinylbenzene) cross-linking improves heat resistance compared with polybutadiene-based systems. Pure ODV still requires blending with OPE-2st for M9-grade laminates such as Panasonic MEGTRON 9. To further enhance thermal stability, fused-ring systems [13] and acenaphthylene (EX)–ODV blends [12,14,15,16] were explored, with EX primarily serving to improve the heat resistance of ODV despite its higher degree of conjugation. Another promising styrene-type resin, 1,2-bis(vinylphenyl)ethane (BVPE) [17,18,19,20,21], can lower the curing temperature of allyl PPO and is increasingly favored by Chinese CCL manufacturers.

BCB resins and derivatives. Benzocyclobutene (BCB) resins deliver superior thermal and dimensional stability through ring-opening cross-linking. Dow’s CYCLOTENE^®^ series [22] (centered on divinylsiloxane–BCB, DVS-BCB) exhibits *D*f ≈ 0.001 but suffers from a relatively large CTE. Recent derivatives include TVCH-BCB [23] and DS-BCB from Wuhan Disai [24] (mass-produced for M8/M9 CCLs), as well as BCB-terminated PPO [25], fluorene-based BCB [26], indene-based BCB oligomers [27], and three-arm BCBs that demonstrate a *D*k as low as 1.83 with a CTE matched to that of copper [28].

Challenges and this work. Despite their advantages, BCB resins suffer from high synthesis costs and a curing-temperature mismatch with styrene-based hydrocarbon resins. To reduce costs, blending with other vinyl-containing resins such as SBS is often necessary; however, SBS exhibits poor heat resistance and is prone to phase separation with BCB, leading to degraded mechanical and dielectric properties. To address these limitations, this work reports a novel low-cost dendritic vinyl resin, BP-TVCH [29], which is rich in allyl groups and β-styryl moieties, enabling it to react with BCB resins and to copolymerize with styrene-type resins. After blending BP-TVCH with PPO, promising dielectric and thermal performance was achieved, as validated by a major CCL manufacturer.

## 2. Materials and Methods

### 2.1. Materials

1,2,4-Trivinylcyclohexane (TVCH), 4,4′-dibromobiphenyl (98%), palladium(II) acetate (Pd(OAc)_2_, 98%), anhydrous N,N-dimethylformamide (DMF, 99.8%, water ≤ 50 ppm) and benzocyclobutene (BCB) were purchased from Sigma-Aldrich (Shanghai, China) and used as received. Toluene, styrene, anhydrous magnesium sulfate (MgSO_4_, ≥99.5%), dichloromethane (DCM, ≥99.8%), and methanol (anhydrous, 99.8%) were purchased from Guangzhou Chemical Reagent Factory and used without further purification unless otherwise specified. Tri-potassium phosphate (K_3_PO_4_) was dried under vacuum at 120 °C for 12 h prior to use. Deionized water (18.2 MΩ·cm) was prepared using a Milli-Q system.

### 2.2. Synthesis of BP-TVCH

A mixture of TVCH (1.0 mol, 162 g), 4,4′-dibromobiphenyl (0.5 mol, 156 g), Pd(OAc)_2_ (1.8 mmol, 404 mg, 0.18 mol % relative to TVCH), and pre-dried K_3_PO_4_ (2.8 mol, 594 g) in anhydrous DMF (4.0 L) was stirred at 140 °C under argon for 48 h. After cooling, the reaction mixture was diluted with toluene (2 L) and washed with deionized water (3 × 2 L). The combined organic layer was dried over anhydrous MgSO_4_ and filtered. The crude product was purified by column chromatography on silica gel using toluene as an eluent. The eluent was concentrated by rotary evaporation in a water bath, and the residue was washed with methanol (3 × 1 L). Final drying under oil-pump vacuum at 75 °C to constant weight gave a reddish-brown, viscous solid (195.2 g, 82% yield based on the oligomeric product).

### 2.3. CCL Fabrication

Copper-clad laminates (CCLs) were prepared at Wazam New Materials Co., Ltd. (Hangzhou, China). A resin formulation comprising BP-TVCH, OPE-2st, complementary resins, and inorganic fillers [30] was dissolved in a suitable solvent (e.g., toluene or cyclohexanone) to prepare a homogeneous varnish. Woven glass fabrics (e.g., 2116 or 1080 style) were impregnated with the varnish, followed by air drying and thermal curing in an oven to produce prepregs with a controlled resin content (typically 30–60 wt%). The prepregs were stacked symmetrically with electrolytic copper foils (e.g., HTE or reverse-treated SI foil) and hot-pressed under vacuum at 180–220 °C and 2–5 MPa to form the final CCLs.

### 2.4. Measurements

Nuclear Magnetic Resonance (NMR). NMR spectra were recorded on a Bruker AVANCE III HD 600 spectrometer.

Thermogravimetric Analysis (TGA). TGA was performed on a TG 209 F3 thermogravimetric analyzer at a heating rate of 10 °C/min under nitrogen.

Differential Scanning Calorimetry (DSC). DSC was conducted on a Mettler Toledo DSC 3 using disposable high-pressure crucibles. Samples were heated from room temperature to 400 °C at 10 °C/min, cooled, and then subjected to a second heating scan to 400 °C at the same rate.

Thermomechanical Analysis (TMA). TMA was conducted on a TA Instruments TMA Q400 thermomechanical analyzer using a two-cycle temperature ramp to eliminate thermal history. The glass transition temperature (Tg) of the CCL samples was measured along the Z axis (thickness direction) according to IPC TM 650 2.4.24.

Dynamic Mechanical Analysis (DMA). DMA was carried out on a TA Discovery DMA 850 under nitrogen using the single-cantilever and three-point bending modes. Temperature sweeps from 30 to 300 °C were performed at 3 °C/min and a fixed frequency of 1 Hz, with strain within the linear viscoelastic region. The glass transition temperature (Tg) was taken from the tan δ peak and cross-checked with the inflection point of the storage modulus (E′) versus temperature plot on a semi log scale.

Dielectric Properties. Dissipation factors (*Df*) at 10 GHz were measured with a Keysight E5071C vector network analyzer according to IPC TM 650 2.5.5.5.

## 3. Results and Discussion

### 3.1. Synthesis of BP-TVCH

**Figure 2:**
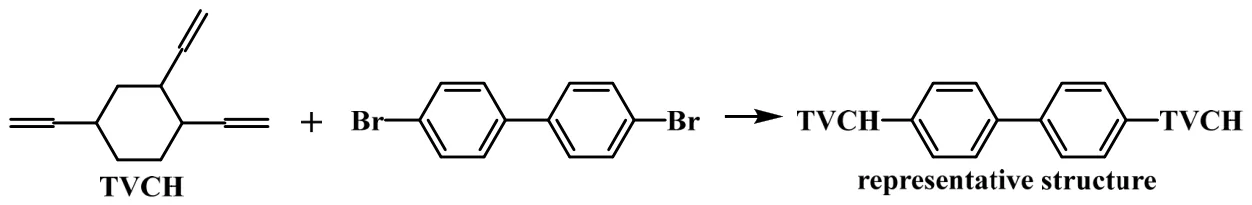
Synthesis of BP-TVCH.

BP-TVCH was synthesized via Heck coupling of TVCH and 4,4′-dibromobiphenyl in a 2:1 molar ratio, as illustrated in Figure 2. The choice of 4,4′-dibromobiphenyl over 1,4-dibromobenzene was critical: the latter underwent incomplete conversion (residual reactant peaks were observed by NMR) and gave a much lower yield. In contrast, 4,4′-dibromobiphenyl afforded a crude yield of 92%, and after purification by column chromatography, a decolorized product was obtained in ~80% isolated yield.

It should be noted that TVCH contains three allyl groups; thus, the theoretical product is not a uniform trimer. For simplicity, it is depicted as a trimer in Figure 2. The reaction may also yield dimers, tetramers, pentamers, and other oligomers, but lower-molecular-weight fractions are readily removed during methanol washing. Furthermore, TVCH itself exists as multiple *cis/trans* isomers, resulting in inherently complex NMR spectra; consequently, the ^1^H-NMR spectrum of BP-TVCH is even more complicated (shown in Figure S1). Nevertheless, the key structural features are clearly identifiable: ^1^H-NMR (600 MHz, CDCl_3_) δ 7.5 (m, Ar–H), 7.4 (m, Ar–H), 6.5–6.2 (m, Ar–CH=CH), 5.8–4.8 (m, RCH=CH_2_), 2.50–0.90 (m, cyclohexyl H). The appearance of the trans-olefinic signal at 6.5–6.2 ppm and aryl H signals at 7.55 and 7.35 ppm confirmed the successful Heck coupling between TVCH and 4,4′-dibromobiphenyl, while the signals at 4.8–5.8 ppm were assigned to the retained vinyl groups. It was found that BP-TVCH was not very stable during storage at room temperature, most likely due to the thermally sensitive β-styryl groups. Because TVCH moieties exhibit complicated spectra, we will elucidate the Heck coupling of alkenes and aryl halides in future studies.

### 3.2. Thermosetting Reaction of BP-TVCH

**Figure 3:**
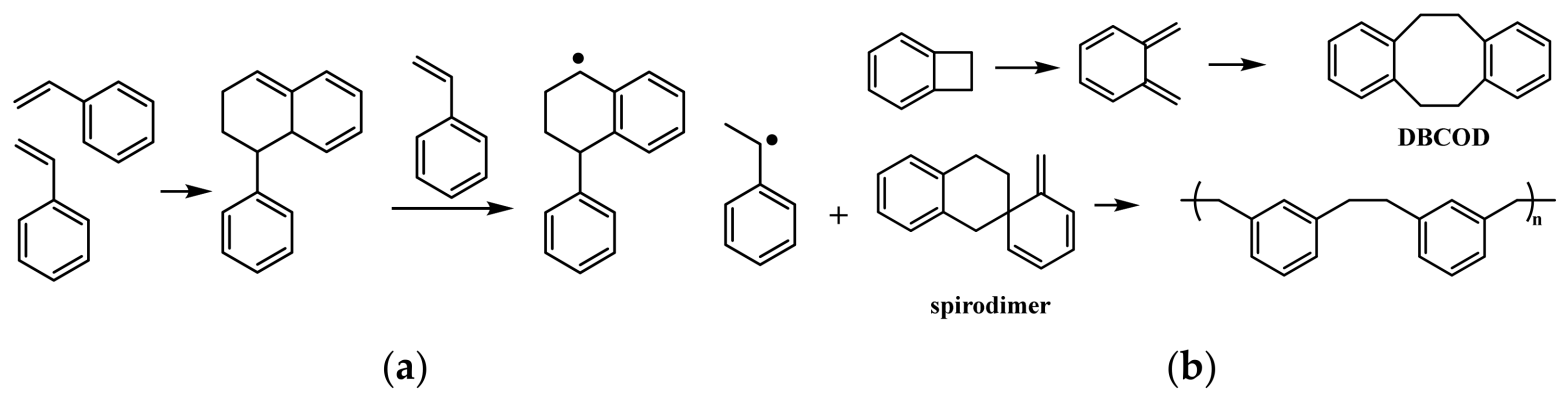
Thermal polymerization/curing mechanism of styrene (**a**) and BCB (**b**).

Currently, thermosetting hydrocarbon resins are mainly classified into two categories based on their crosslinking groups: styrene-based and benzocyclobutene (BCB)-type. Styrene derivatives (including acenaphthylene) undergo free-radical polymerization at elevated temperatures above 100–150 °C. It is generally accepted that the thermal polymerization of styrene involves a Diels–Alder reaction between two styrene groups to form a dimer, which then reacts with a third styrene molecule to generate two types of free radicals [31] (as shown in Figure 3a), thereby initiating the free-radical polymerization of styrene groups to yield a polymer with a polyethylene backbone. In contrast, BCB-type hydrocarbon resins primarily undergo ring-opening at 200–260 °C, initially generating *o*-quinodimethane, followed by the formation of minor amounts of 1,2,5,6-dibenzocyclooctadiene (DBCOD) [32] and a spirodimer [33]. The spirodimer further ring-opens and polymerizes to form poly-*o*-xylylene. BP-TVCH contains both allyl groups and β-styryl moieties, and its curing mechanism requires further investigation.

#### 3.2.1. TGA Curve and DSC Trace of BP-TVCH

**Figure 4:**
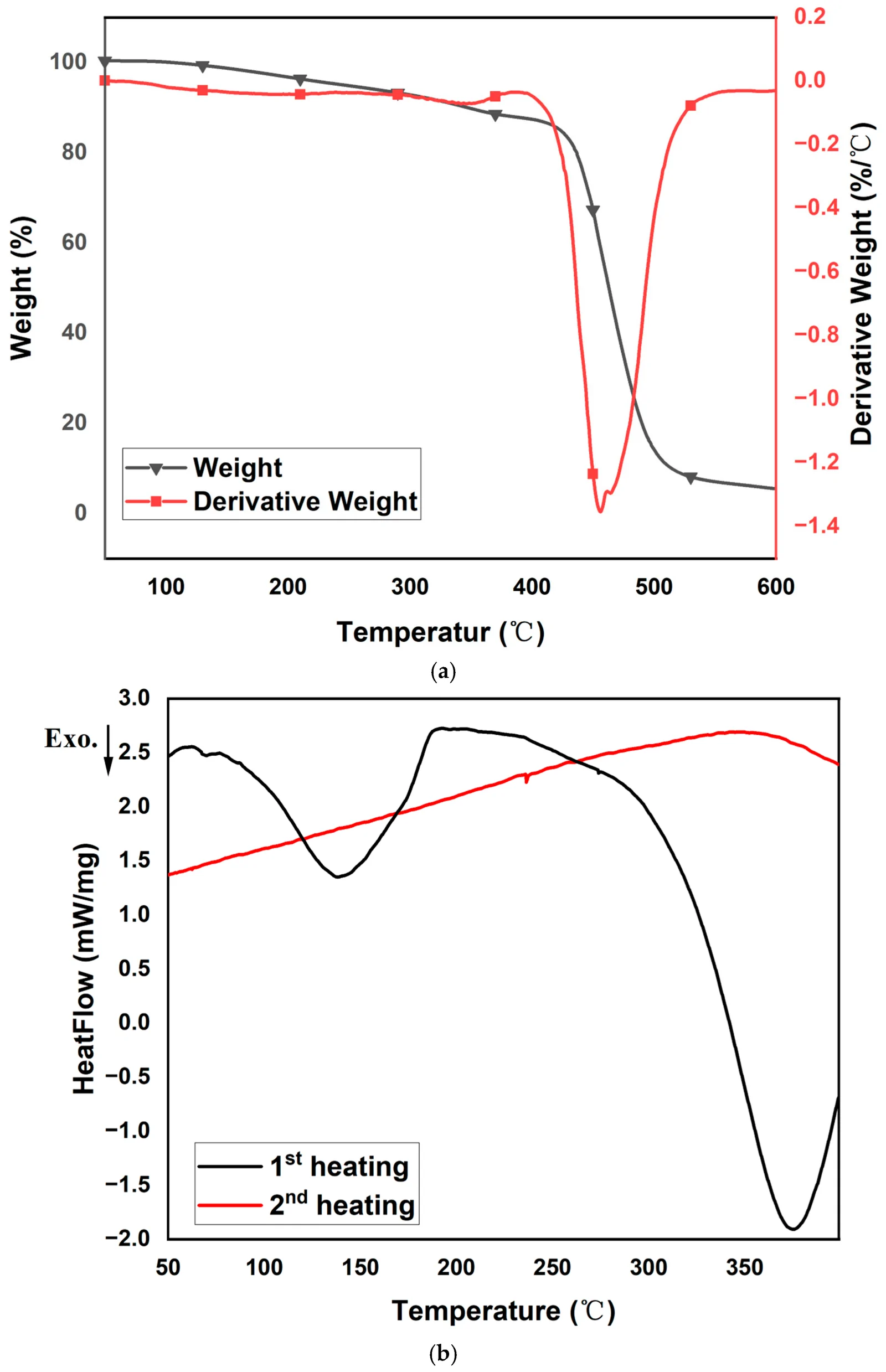
TGA curve (**a**) and DSC trace (**b**) of BP-TVCH.

Figure 4a shows the TGA curve of BP-TVCH recorded at a heating rate of 10 °C/min. Approximately 10% mass loss was observed below 400 °C, which may be attributed partly to slight evaporation or release of volatile low-molecular-weight fractions. Substantial decomposition commenced above 450 °C, indicating that the resin undergoes progressive thermal curing during heating and therefore retains good thermal stability. Above 450 °C, the decomposition rate increased markedly, and the char yield at 600 °C was only about 5%. This suggests predominant alkane/cycloalkane cleavage with little condensed-aromatic (graphitization) formation.

Figure 4b shows the DSC trace of BP-TVCH at 10 °C/min. Because the TGA data indicated some mass loss between 200 and 400 °C, the measurement was performed in disposable high-pressure pans to suppress volatilization. The first heating scan exhibited two major exothermic events: an obvious exotherm centered at ~138 °C, tentatively assignable to homopolymerization/dimerization of the β-styryl moieties, and a broad exotherm around 375 °C, assignable to polymerization involving the allyl groups or/and the further polymerization of β-styryl moieties. Allyl curing typically requires relatively high temperatures; for comparison, the cure exotherm of an allylated indene resin was reported near 320 °C [34] while *trans*-β-methylstyrene (TBMS) even exhibits a distinct high-temperature exothermic peak above 400 °C [35]. In the second heating scan of BP-TVCH, no obvious endothermic or exothermic peaks were observed, indicating that the β-styryl moieties and allyl groups had largely reacted and that no significant depolymerization occurred. It should be noted that the DSC measurement was carried out at 10 °C/min, whereas CCL hot pressing usually employs a staged curing profile (e.g., 200 and 230 °C for several hours); therefore, the DSC results can only guide the design of the lamination process and should not be directly equated with the actual hot-pressing profile.

#### 3.2.2. Gel Content of Cured BP-TVCH

**Figure 5:**
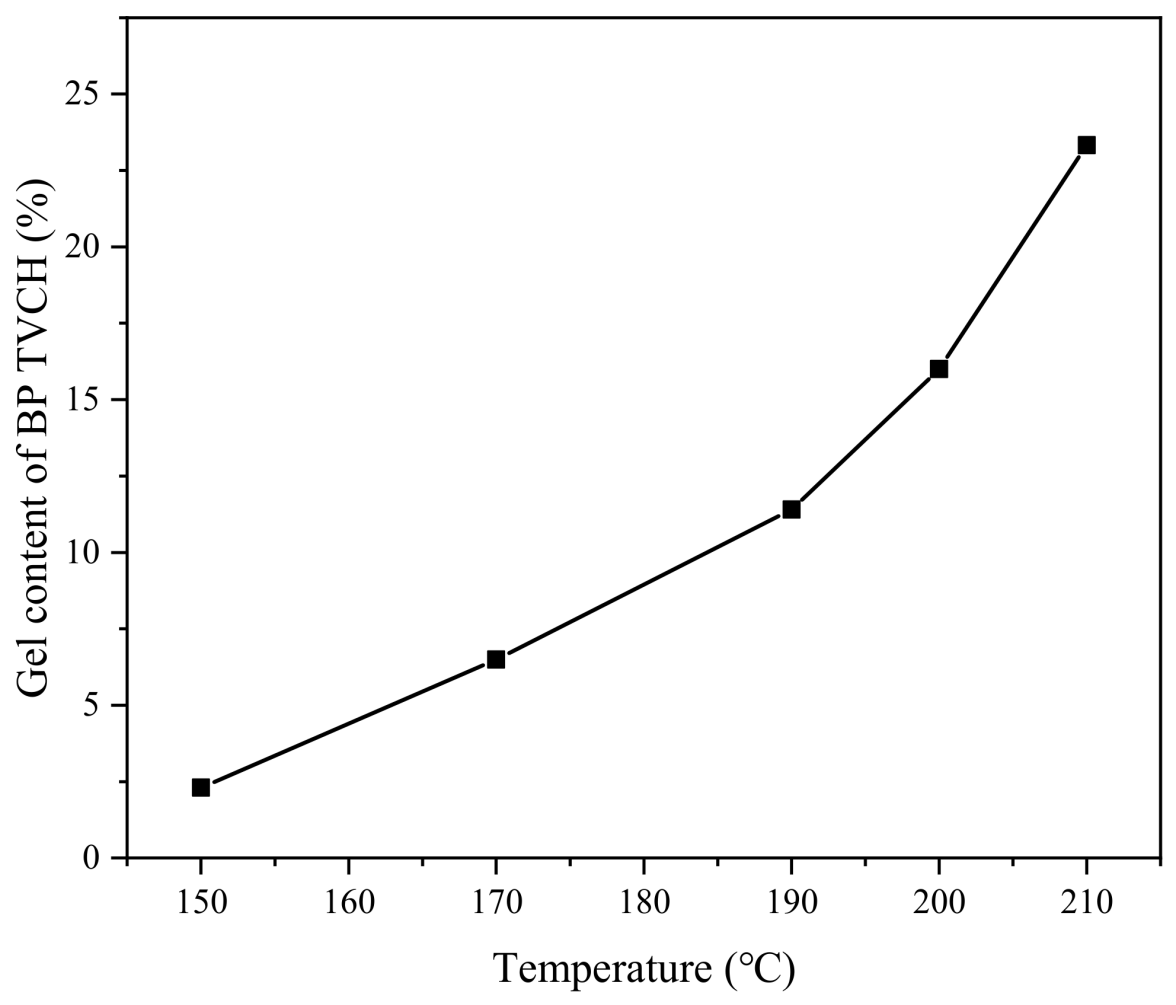
Gel content of BP-TVCH after 6 h heating under nitrogen.

It was found that BP-TVCH is a soluble, viscous soft solid that gradually cures upon heating in the absence of any initiator. As a molecule containing at least four allyl groups and two β-styryl moieties, complete crosslinking would theoretically yield a fully insoluble network. However, the curing rate was found to be relatively slow. Solvent-free BP-TVCH samples were heated at different temperatures for 6 h under nitrogen, followed by exhaustive ultrasonic extraction with dichloromethane (DCM) to determine the insoluble fraction (gel content), as shown in Figure 5. The insoluble fraction was only 2.3% after 6 h at 150 °C, increased to 23.3% at 210 °C, and reached nearly complete insolubility (≈100%) at 250 °C (all samples heated for 6 h), indicating a high degree of crosslinking at elevated temperatures.

The gel content data were difficult to interpret solely from the DSC trace. Although DSC revealed an exothermic peak at ~138 °C (assigned to β-styryl polymerization; Section 3.2.1), heating at 140 °C for 6 h produced almost no macroscopic gel. To clarify this discrepancy, gel permeation chromatography (GPC) was used to estimate the molecular weight of the product (Figure S2). The GPC trace exhibits a bimodal distribution: the main peak (retention time ~13.3 min) corresponds to the higher-molecular-weight fraction, while the secondary peak (~13.8 min) is consistent with lower-molecular-weight oligomers partially retained after methanol washing. The peak at ~14.5 min in Figure S2 should be attributed to an instrumental artifact/solvent peak and is not related to the sample. Quantitative analysis gave *M*_n_ = 1728 Da (Ð = 1.208) for the major distribution and *M*_n_ = 678 Da (Ð = 1.029) for the minor one, collectively confirming the oligomeric nature of the product. These results indicate that BP-TVCH undergoes significant polymerization at 140 °C, but the resulting molecular weight remains insufficient to form a cross-linked gel network, accounting for its solubility.

#### 3.2.3. Reactivity Study of Allyl Groups

To clarify the distinct roles of allyl groups in BP-TVCH during thermal curing, the reactivities of various model compounds were evaluated using 4,4′-dimethylbiphenyl as an internal standard to estimate the extent of conversion. The results are summarized in Table 1, and the corresponding NMR spectra are provided in the Supporting Information (Figures S3–S8).

It should be noted that because the model compounds used—TVCH, BCB, and styrene—are relatively volatile, reactions were conducted in screw-cap headspace vials to prevent loss of material. Additionally, 4,4′-dimethylbiphenyl exhibits poor solubility in TVCH at room temperature; therefore, the mixture was gently warmed prior to sampling, and deuterated chloroform (CDCl_3_) was added immediately for NMR analysis. After the reaction, the vials were allowed to cool thoroughly. Deuterated chloroform was then injected through the septum of the cap to dilute the reaction mixture. After complete dissolution, the vial was opened for sampling. This procedure was critical to minimize volatilization and ensure reliable quantitative data.

First, it was found that pure TVCH heated at 180 °C for 20 h showed almost no reaction, while heating at 210 °C for 20 h resulted in only a 3.6% decrease in the allylic vinylic proton integration. This indicates that the curability of BP-TVCH is not attributable to the allyl groups but rather to the β-styryl moieties. However, when TVCH and styrene were heated together at 180 °C for 20 h, a viscous liquid was obtained, which solidified into a soft solid upon cooling and was completely soluble in CDCl_3_. ^1^H NMR comparison revealed that the allylic vinylic proton integration decreased by ~60%, while ~3–4% of styrene remained. This demonstrates that TVCH can participate in styrene polymerization. Although TVCH hardly homopolymerizes below 210 °C, its allyl groups exhibit significant chain-transfer characteristics in the presence of active radicals. However, the resulting allylic radicals have low reactivity, preventing the formation of high-molecular-weight polymers and leaving a small amount of unreacted styrene monomer.

For unsubstituted BCB heated at 180 °C for 20 h, the benzocyclobutene ring proton integration decreased by only 3.5%; at 210 °C for 20 h, it decreased by 52%. The formation of DBCOD via [4+4] cyclization [32] was observed, with the benzocyclobutene ring proton signal located at δ 3.06. When BCB and styrene were heated at 180 °C for 20 h, a hard solid was obtained that was fully soluble in CDCl_3_. ^1^H NMR comparison showed that the cyclobutene ring proton integration decreased by ~12%, while almost no residual styrene peaks were detectable. This indicates that although BCB and styrene have mismatched polymerization windows, they still undergo partial polymerization, although it cannot be determined whether true copolymerization occurred.

When BCB and TVCH were heated at 210 °C for 20 h, a semisolid product was obtained. The allylic vinylic proton integration decreased by 16% (compared to only 3.6% for pure TVCH under the same conditions), and the benzocyclobutene ring proton integration decreased by 36% (lower than the 52% observed for pure BCB at 210 °C), with no characteristic DBCOD [32] peaks observed. It can therefore be concluded that BCB primarily undergoes ring-opening polymerization to form poly-*o*-xylylene [33], while TVCH acts as a chain-transfer agent, participating in the reaction and increasing its own conversion. Concurrently, the conversion of BCB is suppressed, and the [4+4] cyclization pathway is inhibited.

### 3.3. Performance of BP TVCH in Copper Clad Laminates (CCLs)

Although polyphenylene ether (PPO) typically exhibits a dielectric loss of ~0.002, its excellent thermal resistance and mechanical properties make it indispensable in M8 and M9 high-speed laminates. BP-TVCH is synthetically straightforward and cost-effective: its β-styryl moieties enable self-curing, while the allyl groups can copolymerize with styrene-based and BCB resins. Therefore, BP-TVCH was selected as an additive to blend with OPE-2st. CCLs were fabricated at Wazam New Materials, and their key properties are summarized in Table 2.

#### 3.3.1. Interfacial Adhesion

The CCL demonstrated robust interfacial bonding between the resin and copper foil. For HTE (high-temperature electrolytic) copper foil, the peel strength was 0.280 N/mm at room temperature and 0.290 N/mm at elevated temperature. For SI copper foil, the values were 0.190 N/mm (room temperature) and 0.180 N/mm (elevated temperature). After thermal treatment, the peel strength showed no significant decline, confirming stable resin/copper interfacial adhesion. Additionally, the interlayer peel strength was 0.093 N/mm, indicating adequate bonding stability between prepreg layers. In summary, the CCL exhibits a stable copper-foil/resin interface without noticeable strength degradation after thermal exposure.

#### 3.3.2. Thermal Dimensional Stability

The CTE was measured on a TA Instruments TMA Q400 (see Experimental Section; spectra in Figure S9–S11). The in-plane CTE (X/Y-CTE, 50–125 °C) was 10.93 and 10.96 ppm/°C, respectively, indicating good isotropic thermal expansion. The out-of-plane Z-CTE (α1) was 16.33 ppm/°C. Furthermore, the Z-directional dimensional change rate between 50 and 260 °C was only 0.4762%. These results demonstrate excellent thermal dimensional stability over a broad temperature range. For CCLs, a low Z-CTE is particularly advantageous as it mitigates thermal stress induced by CTE mismatch among the resin, copper, and glass fiber during thermal cycling and high-temperature PCB processing. We attribute the excellent dimensional stability to the multi-functional crosslinkable architecture of BP-TVCH. Unlike conventional bifunctional resins (e.g., standard epoxy or bismaleimide systems), BP-TVCH bears multiple reactive sites—including several β-styryl moieties and allyl groups—per molecule, enabling a higher crosslinking density and a more rigid three-dimensional network upon curing. This densely crosslinked structure effectively restricts chain segment mobility, resulting in the observed low in-plane and out-of-plane CTE values.

#### 3.3.3. Thermomechanical Properties

The material exhibited a high glass transition temperature (Tg). DMA testing (Figures S12 and S13) yielded Tg values of 277.58 °C (single-cantilever mode), 246.17 °C (three-point bending mode), and 260.23 °C (CTE method).

The storage modulus (E′) was also remarkable:

• Single-cantilever: 14.63 GPa at 50 °C, retaining 8.80 GPa at 250 °C;
• Three-point bending: 20.72 GPa at 50 °C, retaining 13.01 GPa at 250 °C.

This indicates that the material maintains high stiffness from 50 to 250 °C. Notably, under three-point bending, E′ at 250 °C still reached 13.01 GPa, demonstrating strong high-temperature structural stability of the crosslinked network. This combination of high Tg and excellent high-temperature modulus retention is one of the most noteworthy performance advantages of this system, which we attribute to the rigid biphenyl backbone and the multi-functional crosslinking architecture of BP-TVCH. The rigid aromatic core restricts segmental motion, while the high crosslink density suppresses viscous flow, collectively contributing to both the elevated Tg and the exceptional modulus retention at elevated temperatures.

#### 3.3.4. Dielectric Properties

At 10 GHz and a thickness of 0.135 mm, the CCL showed a dielectric loss (*Df*) of 0.00101 initially, with only a marginal rise to 0.00105 after 1 h, which remained stable through 24 h, confirming low and stable high-frequency dielectric loss. This ultra-low *Df* is attributed to the fully hydrocarbon structure of BP-TVCH, which contains no heteroatoms and thus minimizes dipole polarization. When blended with OPE-2st, the nonpolar hydrocarbon network further dilutes the residual polarity of the PPO matrix, reducing the overall *Df* below that of PPO alone (~0.002). The stable *Df* over 24 h indicates good compatibility between the two phases, with minimal interfacial polarization loss.

## 4. Discussion

A novel multi-functional hydrocarbon resin, BP-TVCH, was synthesized from biphenyl and 1,2,4-trivinylcyclohexane, bearing both β-styryl moieties and allyl groups.

Curing proceeds via two pathways: β-styryl moieties undergo addition/coupling reactions at ~138 °C, resulting in a molecular weight increase without immediate gelation, while allyl groups contribute to high-temperature cross-linking through chain-transfer reactions rather than acting as primary crosslinking sites.

The multi-functional architecture enables a densely crosslinked network with excellent dimensional stability (X/Y-CTE ~10.9 ppm/°C, Z-CTE 16.3 ppm/°C) and high Tg (up to 277 °C).

The fully hydrocarbon, heteroatom-free structure delivers ultra-low dielectric loss (*Df* = 0.00101–0.00105 at 10 GHz) when blended with OPE-2st, outperforming conventional PPO-based systems.

The chain-transfer characteristic of allyl groups suggests potential compatibility of BP-TVCH with styryl and BCB-based resins, which may provide a useful direction for future development of high-frequency CCL materials.

## Figures and Tables

**Table 1:** Reactivity of model compounds under different conditions.

Reactant		Temperature * Time	Monitored Group	Conversion (%)	NMR Spectra
TVCH		210 °C*20 h	allylic vinyl	3.6%	Figure S3
TVCH	styrene	180 °C*20 h	allylic vinyl	60%	Figure S4
			styryl vinyl	~96%	Figure S4
BCB		180 °C*20 h	cyclobutene	3.5%	Figure S5
BCB		210 °C*20 h	cyclobutene	52%	Figure S6
BCB	styrene	180 °C*20 h	cyclobutene	12%	Figure S7
			styryl vinyl	>99%	Figure S7
TVCH	BCB	210 °C*20 h	allylic vinyl	16%	Figure S8
			cyclobutene	36%	Figure S8

**Table 2:** Main performance of CCLs from BP TVCH and OPE-2st.

Property	Item	Value	
Peeling strength (Hoz)	ambient	0.280 N/mm (HTE *)	0.190 N/mm (SI *)
	thermal treatment	0.290 N/mm (HTE *)	0.180 N/mm (SI *)
	interlayer	0.093 N/mm	
CTE thk:1.2311 mm	X-CTE(50–125 °C)	10.93 ppm/°C	
	Y-CTE(50–125 °C)	10.96 ppm/°C	
	Z-CTE (α1)	16.33 ppm/°C	
	Total Z-CTE	0.4762%	
DMA(SC) thk:1.23 mm	Storage Modulus (E’) 50 °C	14.6254 GPa	
	Storage Modulus (E’) 250 °C	8.8035 GPa	
DMA (TPB) thk:1.27 mm	Storage Modulus (E’) 50 °C	20.7222 GPa	
	Storage Modulus (E’) 250 °C	13.0126 GPa	
Tg	DMA (SC)	277.58 °C	
	DMA(TPB)	246.17 °C	
	CTE	260.23 °C	
Tanδ *Df* (10 G) thk: 0.135 mm	initial	0.00101	
	1 h	0.00105	
	24 h	0.00105	

* Note: HTE: a rougher copper foil, SI: a smoother copper foil, SC: Single Cantilever, TPB: Three-point bending, thk: thickness.

## Data Availability

The data presented in this study are available from the corresponding author on reasonable request.

## Supplementary Materials

The following supporting information can be downloaded at: https://www.mdpi.com/article/10.3390/polym18212672/s1, Figure S1. ^1^H NMR spectra of BP-TVCH. Figure S2. GPC trace of BP TVCH after heating at 140 °C for 6 h. Figure S3. ^1^H NMR spectra of TVCH before (top) and after heating at 210 °C for 20 h (bottom), with 4,4′-dimethylbiphenyl as an internal standard. The allylic vinylic H signal decreased by approximately 3.6%. Figure S4. ^1^H NMR spectra of a ~1:1 molar mixture of TVCH and styrene before (top) and after heating at 180 °C for 20 h (bottom), with 4,4′-dimethylbiphenyl as an internal standard. The allylic vinylic H signal decreased by approximately 60%, while styrene conversion exceeded 96%. Figure S5. ^1^H NMR spectra of BCB before (top) and after heating at 180 °C for 20 h (bottom), with 4,4′-dimethylbiphenyl as an internal standard. The cyclobutene signal decreased by approximately 3.5%. Figure S6. ^1^H NMR spectra of BCB before (top) and after heating at 210 °C for 20 h (bottom), with 4,4′-dimethylbiphenyl as an internal standard. The cyclobutene signal decreased by approximately 52%. Figure S7. ^1^H NMR spectra of a ~1:1 molar mixture of BCB and styrene before (top) and after heating at 180 °C for 20 h (bottom), with 4,4′-dimethylbiphenyl as an internal standard. The cyclobutene signal decreased by approximately 12%, while styrene conversion exceeded 99%. Figure S8. ^1^H NMR spectra of a ~1:1 molar mixture of BCB and TVCH before (top) and after heating at 210 °C for 20 h (bottom), with 4,4′-dimethylbiphenyl as an internal standard. The cyclobutene signal decreased by approximately 36%, while the allylic vinylic H signal decreased by approximately 36%. Figure S9. X CTE of CCL from BP-TVCH /OPE-2st blends, measured by TA Instruments TMA Q400 according to IPC TM 650 2.4.24. Figure S10. Y CTE of CCL from BP-TVCH /OPE-2st blends, measured by TA Instruments TMA Q400 according to IPC TM 650 2.4.24. Figure S11. Z CTE of CCL from BP-TVCH /OPE-2st blends, measured by TA Instruments TMA Q400 according to IPC TM 650 2.4.24. Figure S12. DMA curves of BP TVCH/OPE-2st CCLs measured by single cantilever mode on a TA Discovery DMA 850 under nitrogen flow (1 Hz, heating rate 3 °C/min, 30–300 °C). Figure S13. DMA curves of BP TVCH/OPE-2st CCLs measured by three-point bending mode on a TA Discovery DMA 850 under nitrogen flow (1 Hz, heating rate 3 °C/min, 30–300 °C).
