# Supplementary material for: Cross-Linkable Hydrocarbon Resins Bearing β-Styryl Moieties and Allyl Groups: Synthesis, Curing Behavior and Performance for Copper-Clad Laminate Applications
Source: Polymers (Basel). 2026 Oct 28;18(21):2672. doi: 10.3390/polym18212672 (PMC13765719; doi:10.3390/polym18212672)
Supplement: Supplementary file 1 [file polymers-18-02672-s001.zip › polymers-4616896-supplementary.pdf]

Cross Linkable Hydrocarbon Resins Bearing β-Styryl moieties and Allyl groups: Synthesis, Curing Behavior and Performance for CCL Applications

Zhaoji Li and Yueqi Mo *

School of Materials Science and Engineering, South China University of Technology, Guangzhou, Guangdong 510640, China. 1391916776@qq.com; pomoy@scut.edu.cn

Correspondence: pomoy@scut.edu.cn

Contents

Figure S1. ^{1}H NMR spectra of BP-TVCH---**2**

Figure S2. GPC trace of BP-TVCH after heating at 140 °C for 6 h. -----------------------------------**2**

Figure S3. ^{1}H NMR spectra of TVCH before (top) and after heating at 210 °C for 20 h (bottom), with 4,4′-dimethylbiphenyl as an internal standard. --**3**

Figure S4. ^{1}H NMR spectra of a ~1:1 molar mixture of TVCH and styrene before (top) and after heating at 180 °C for 20 h (bottom), with 4,4′-dimethylbiphenyl as an internal standard. -----------**4**

Figure S5. ^{1}H NMR spectra of BCB before (top) and after heating at 180 °C for 20 h (bottom), with 4,4′-dimethylbiphenyl as an internal standard. --**5**

Figure S6. ^{1}H NMR spectra of BCB before (top) and after heating at 210 °C for 20 h (bottom), with 4,4′-dimethylbiphenyl as an internal standard. --**6**

Figure S7. ^{1}H NMR spectra of a ~1:1 molar mixture of BCB and styrene before (top) and after heating at 180 °C for 20 h (bottom), with 4,4′-dimethylbiphenyl as an internal standard. -----------**7**

Figure S8. ^{1}H NMR spectra of a ~1:1 molar mixture of BCB and TVCH before (top) and after heating at 210 °C for 20 h (bottom), with 4,4′-dimethylbiphenyl as an internal standard. -----------**8**

Figure S9. X-CTE of CCL from BP-TVCH /OPE-2st blends, measured by TA Instruments TMA Q400 according to IPC-TM-650 2.4.24. --**9**

Figure S10. Y-CTE of CCL from BP-TVCH /OPE-2st blends, measured by TA Instruments TMA Q400 according to IPC-TM-650 2.4.24. --**9**

Figure S11. Z-CTE of CCL from BP-TVCH /OPE-2st blends, measured by TA Instruments TMA Q400 according to IPC-TM-650 2.4.24. ---**10**

Figure S12. DMA curves of BP TVCH/OPE-2st CCLs measured by single-cantilever mode on a TA Discovery DMA 850 under nitrogen flow (1 Hz, heating rate 3 °C/min, 30–300 °C). ---------**10**

Figure S13. DMA curves of BP TVCH/OPE-2st CCLs measured by three-point bending mode on a TA Discovery DMA 850 under nitrogen flow (1 Hz, heating rate 3 °C/min, 30–300 °C). **-------11**

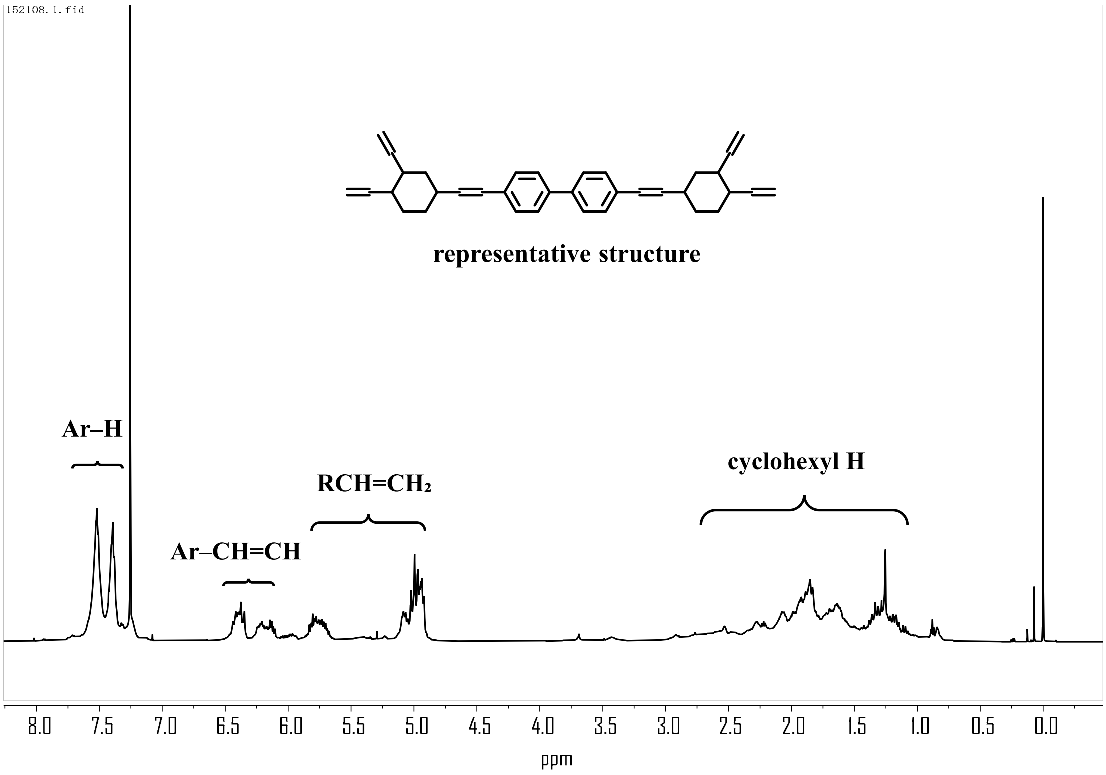

Figure S1. ^{1}H NMR spectra of BP-TVCH

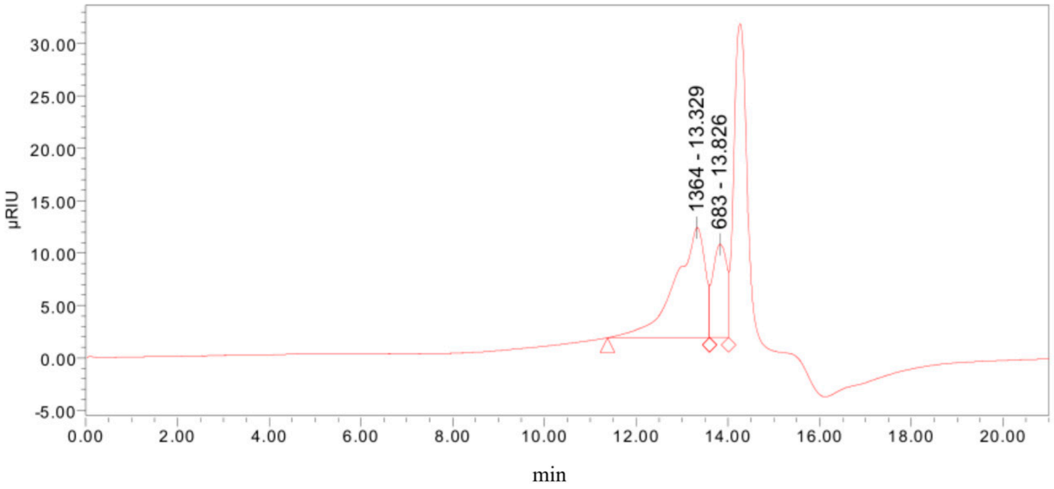

Distribution Name	Mn (Daltons)	Mw (Daltons)	MP	Mz (Daltons)	Mz+1 (Daltons)	Polydispersity
1	1728	2088	1364	2653	3416	1.208252
2	678	698	683	718	738	1.029185

Figure S2. GPC trace of BP-TVCH after heating at 140 °C for 6 h.

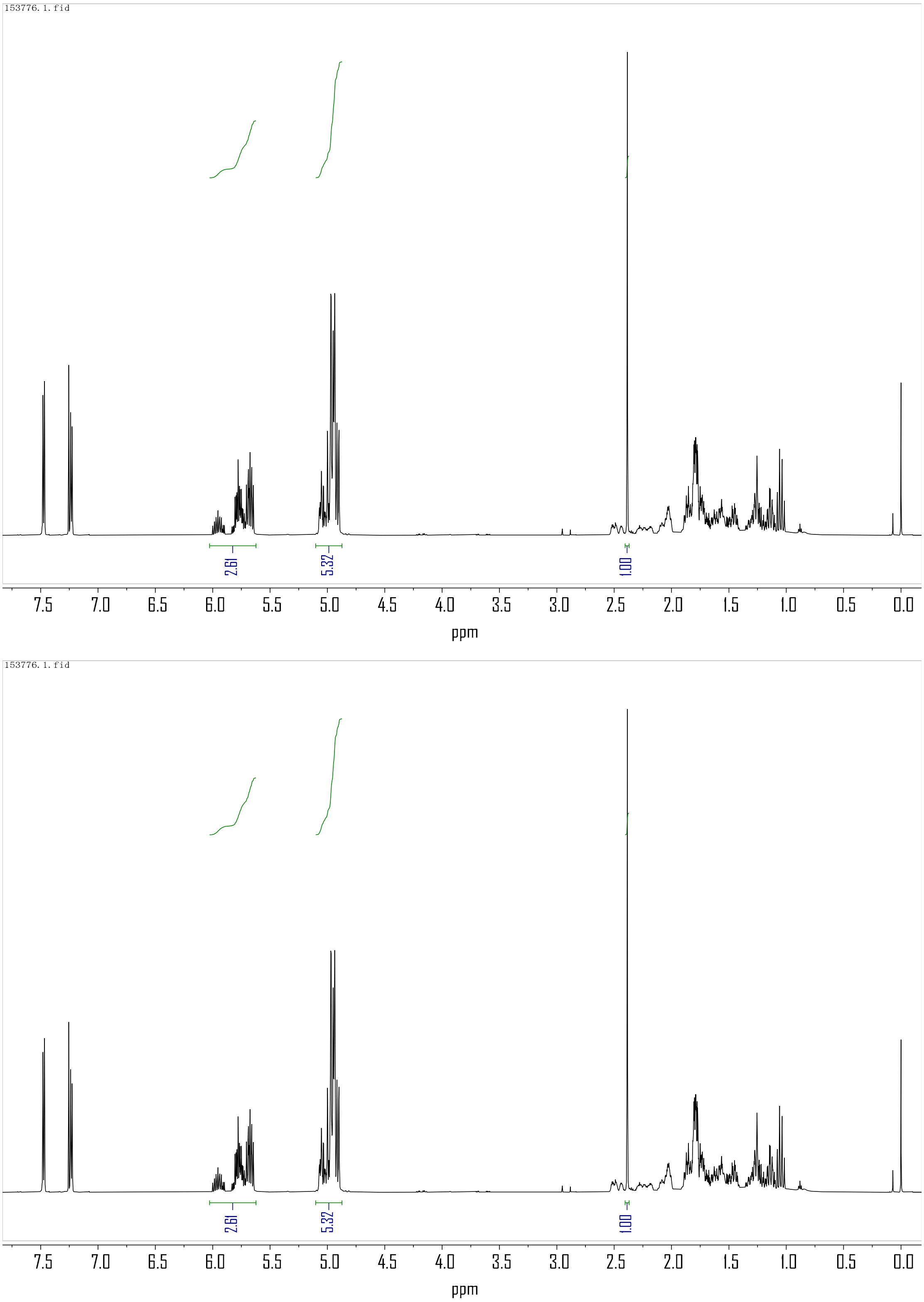

Figure S3. ^{1}H NMR spectra of TVCH before (top) and after heating at 210 °C for 20 h (bottom), with 4,4′-dimethylbiphenyl as an internal standard. The allylic vinylic H signal decreased by approximately 3.6%.

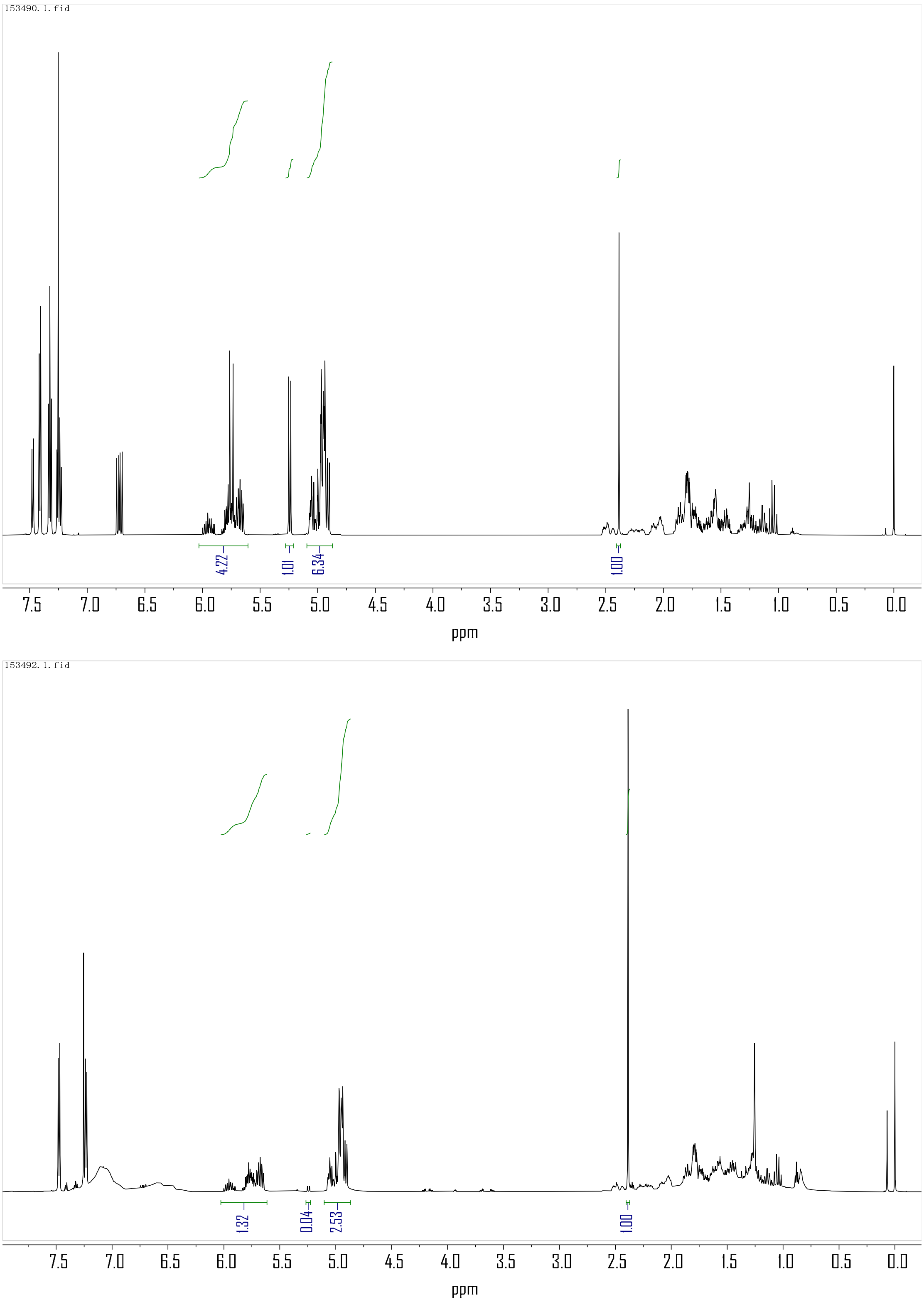

Figure S4. ^{1}H NMR spectra of a ~1:1 molar mixture of TVCH and styrene before (top) and after heating at 180 °C for 20 h (bottom), with 4,4′-dimethylbiphenyl as an internal standard. The allylic vinylic H signal decreased by approximately 60%, while styrene conversion exceeded 96%.

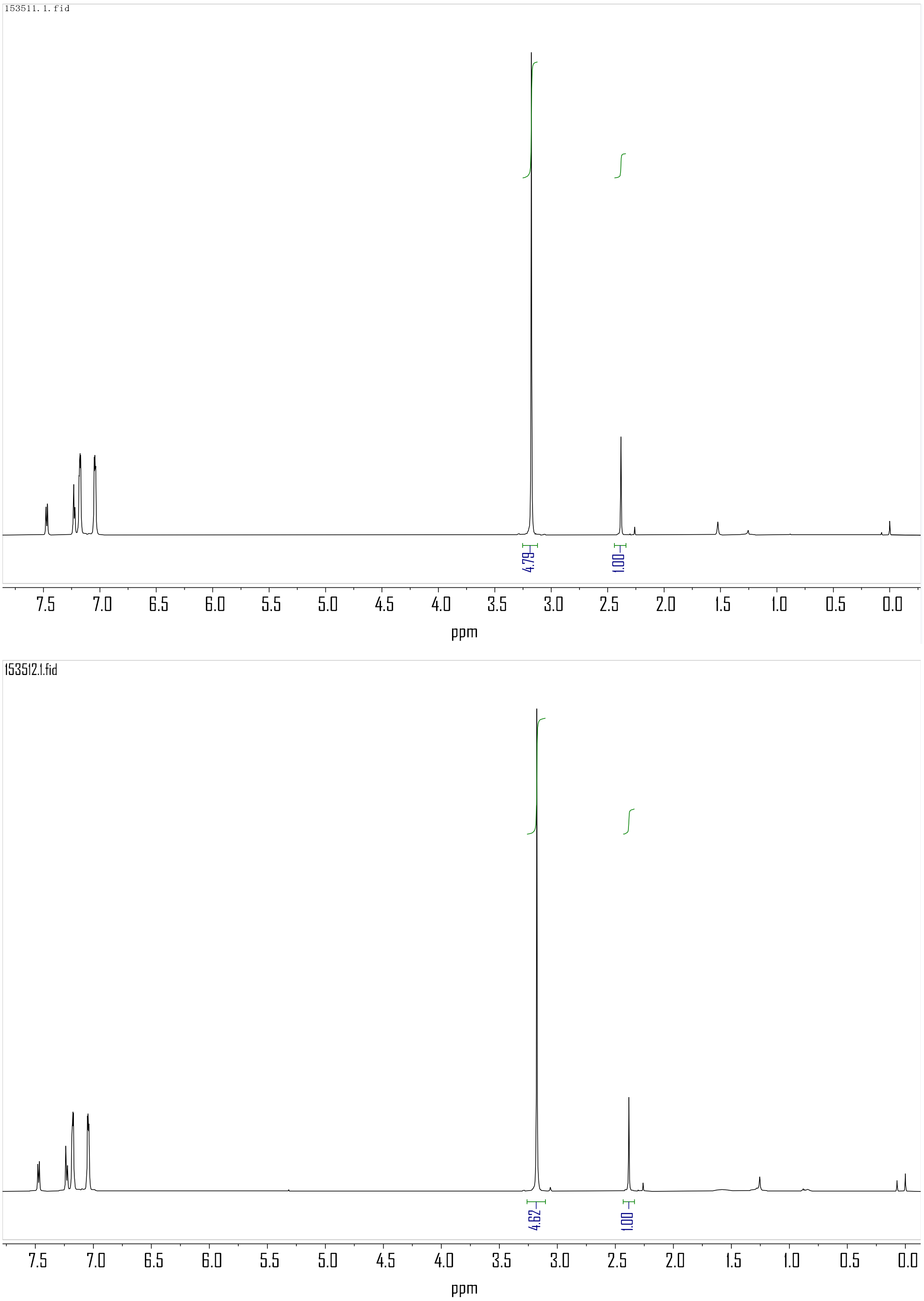

Figure S5. ^{1}H NMR spectra of BCB before (top) and after heating at 180 °C for 20 h (bottom), with 4,4′-dimethylbiphenyl as an internal standard. The cyclobutene signal decreased by approximately 3.5%.

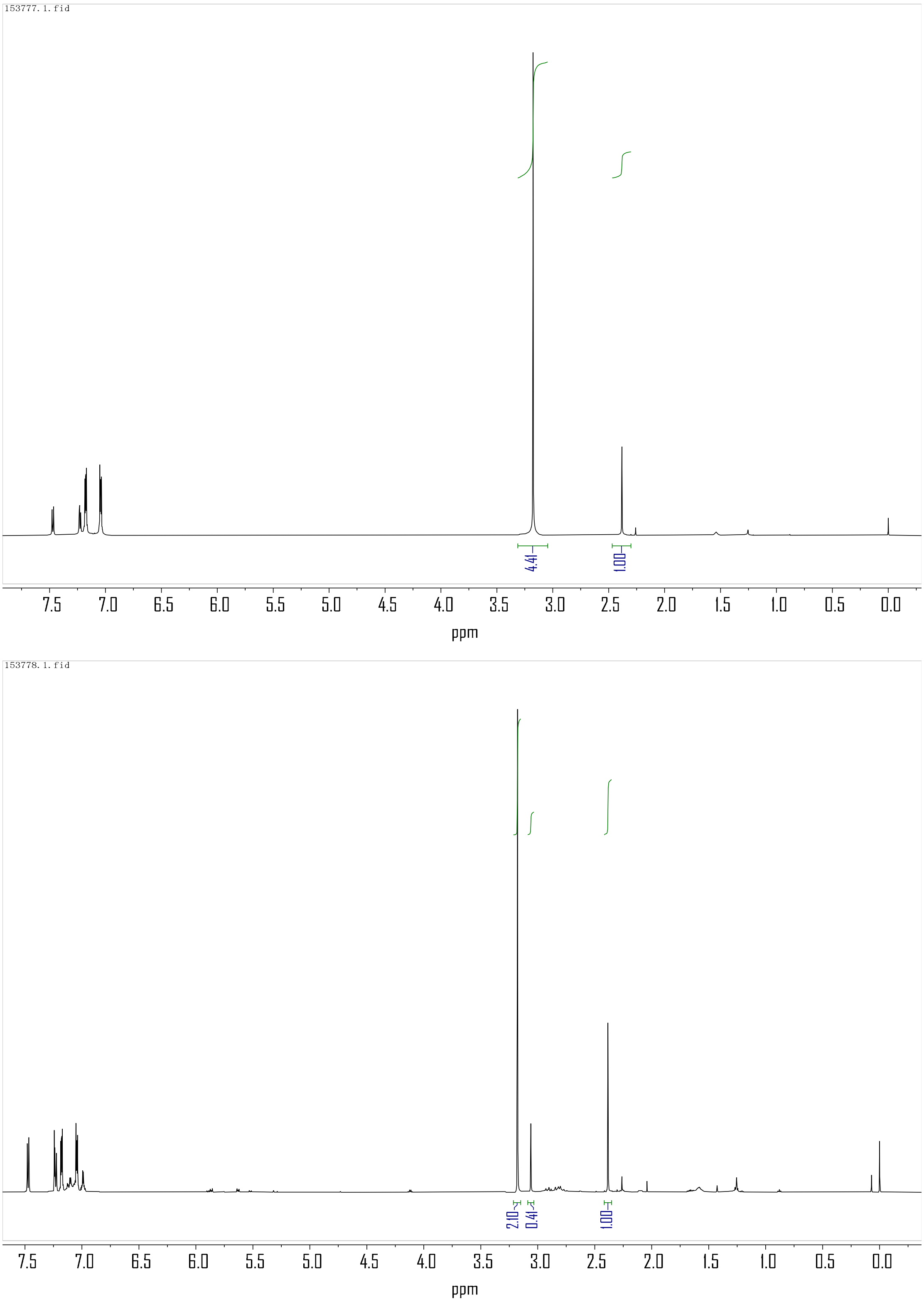

Figure S6. ^{1}H NMR spectra of BCB before (top) and after heating at 210 °C for 20 h (bottom), with 4,4′-dimethylbiphenyl as an internal standard. The cyclobutene signal decreased by approximately 52%.

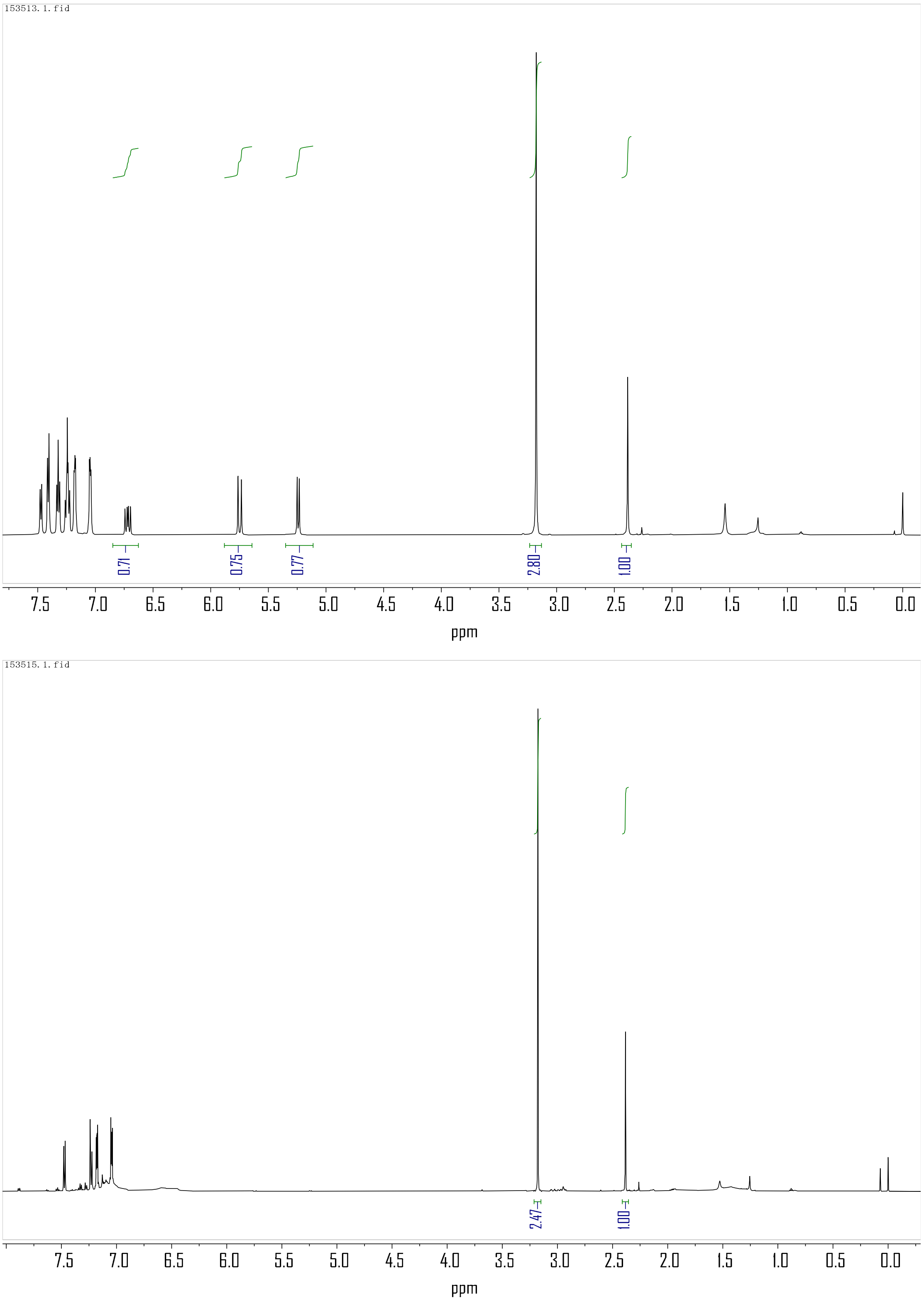

Figure S7. ^1H NMR spectra of a ~1:1 molar mixture of BCB and styrene before (top) and after heating at 180 °C for 20 h (bottom), with 4,4′-dimethylbiphenyl as an internal standard. The cyclobutene signal decreased by approximately 12%, while styrene conversion exceeded 99%.

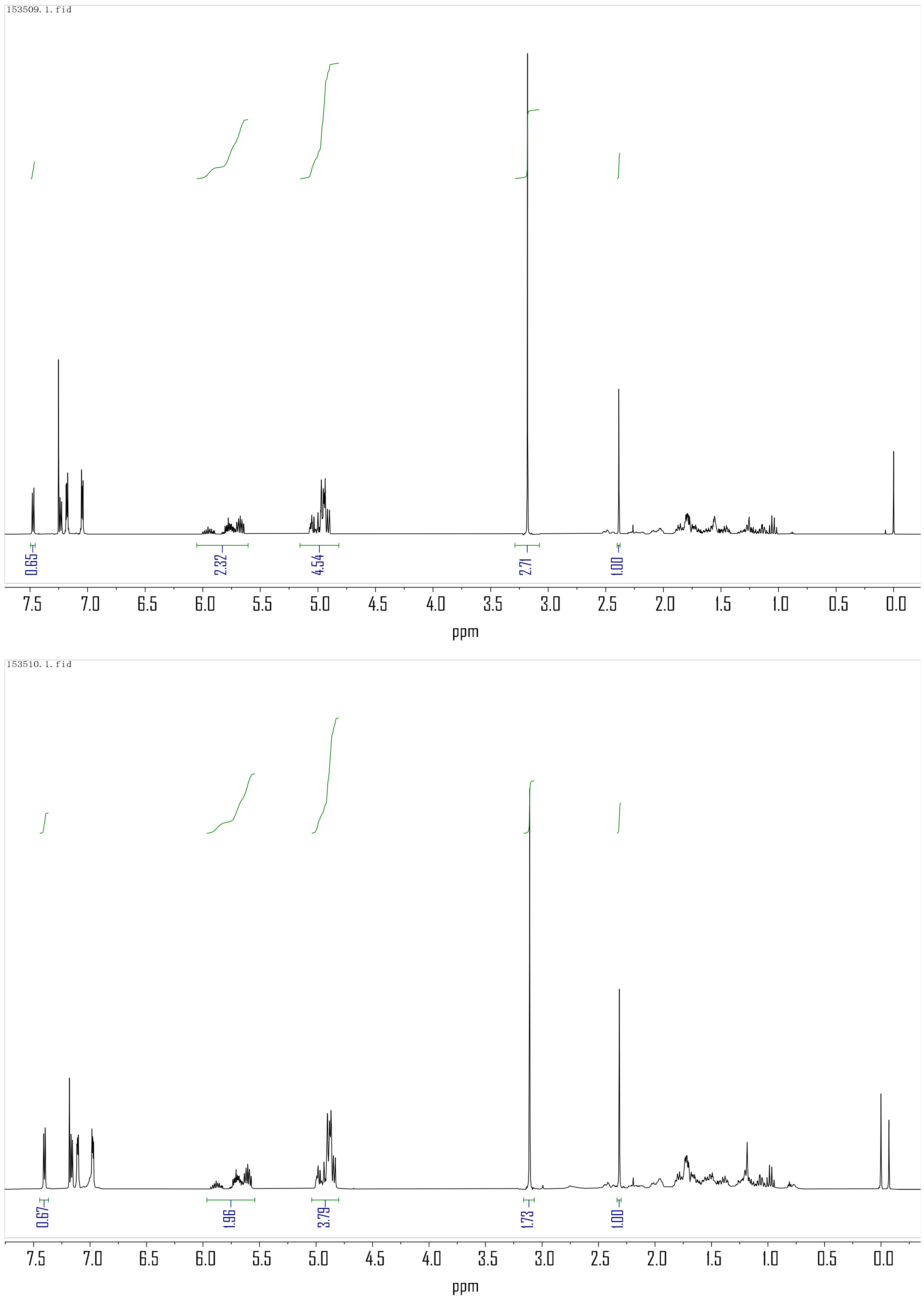

Figure S8. ^1H NMR spectra of a ~1:1 molar mixture of BCB and TVCH before (top) and after heating at 210 °C for 20 h (bottom), with 4,4′-dimethylbiphenyl as an internal standard. The cyclobutene signal decreased by approximately 36%, while the allylic vinylic H signal decreased by approximately 36%.

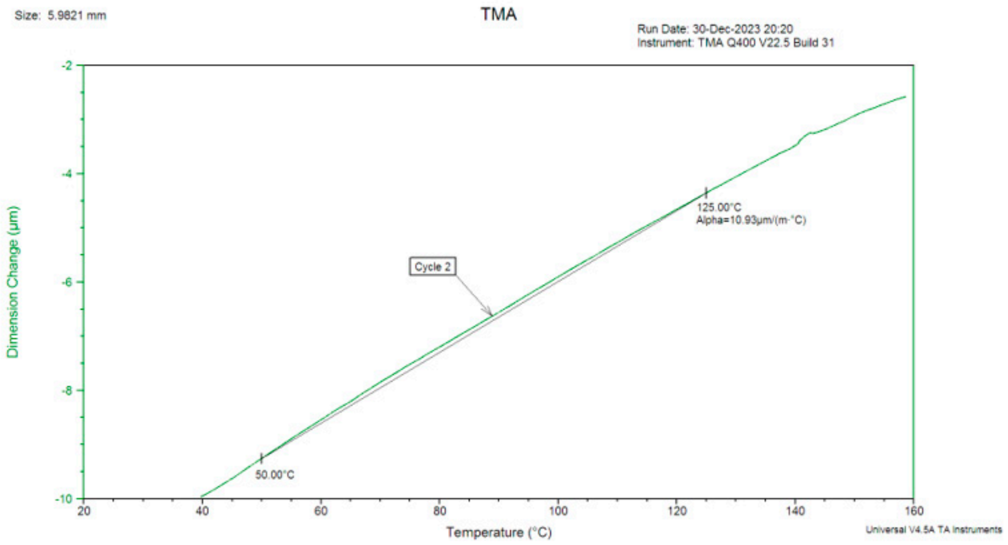

Figure S9. X-CTE of CCL from BP-TVCH /OPE-2st blends, measured by TA Instruments TMA Q400 according to IPC-TM-650 2.4.24.

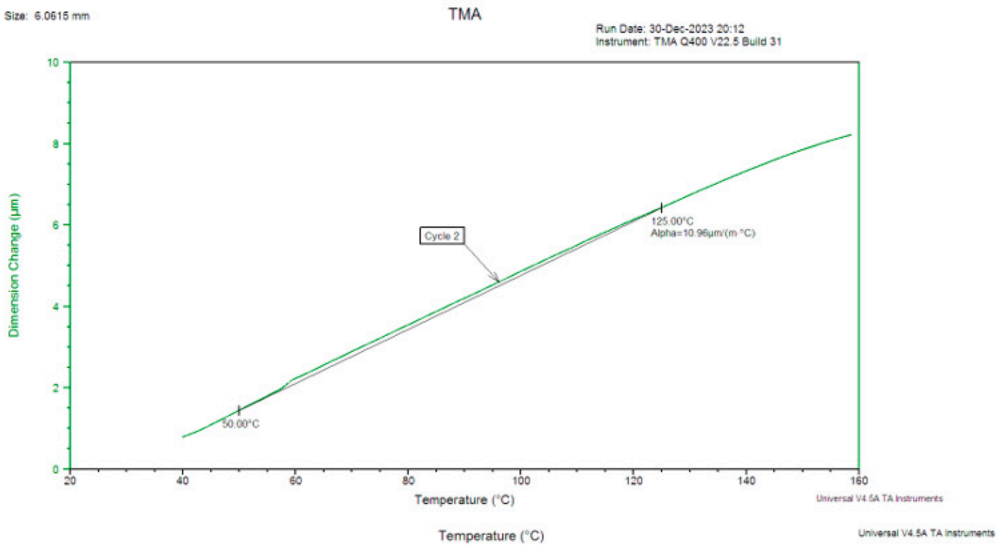

Figure S10. Y-CTE of CCL from BP-TVCH /OPE-2st blends, measured by TA Instruments TMA Q400 according to IPC-TM-650 2.4.24.

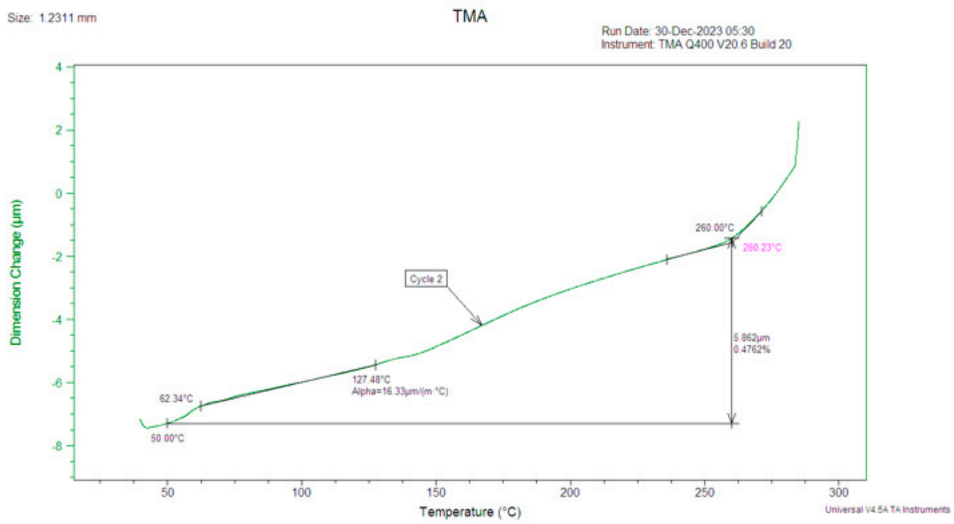

Figure S11. Z-CTE of CCL from BP-TVCH /OPE-2st blends, measured by TA Instruments TMA Q400 according to IPC-TM-650 2.4.24.

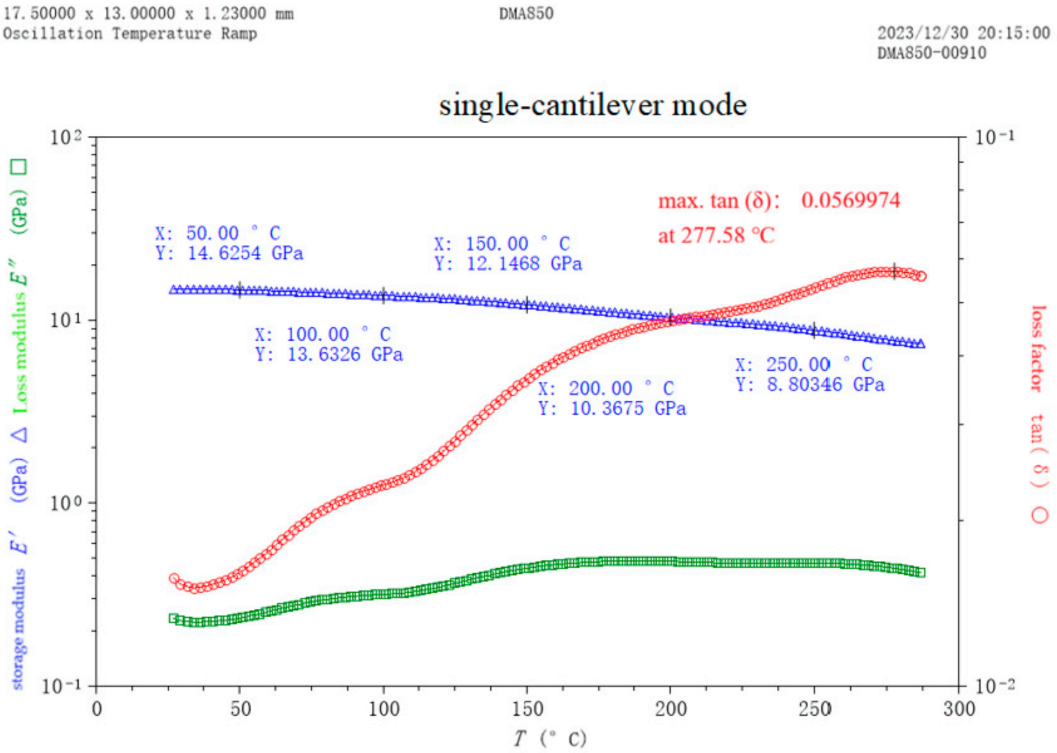

Figure S12. DMA curves of BP TVCH/OPE-2st CCLs measured by single-cantilever mode on a TA Discovery DMA 850 under nitrogen flow (1 Hz, heating rate 3 °C/min, 30–300 °C).

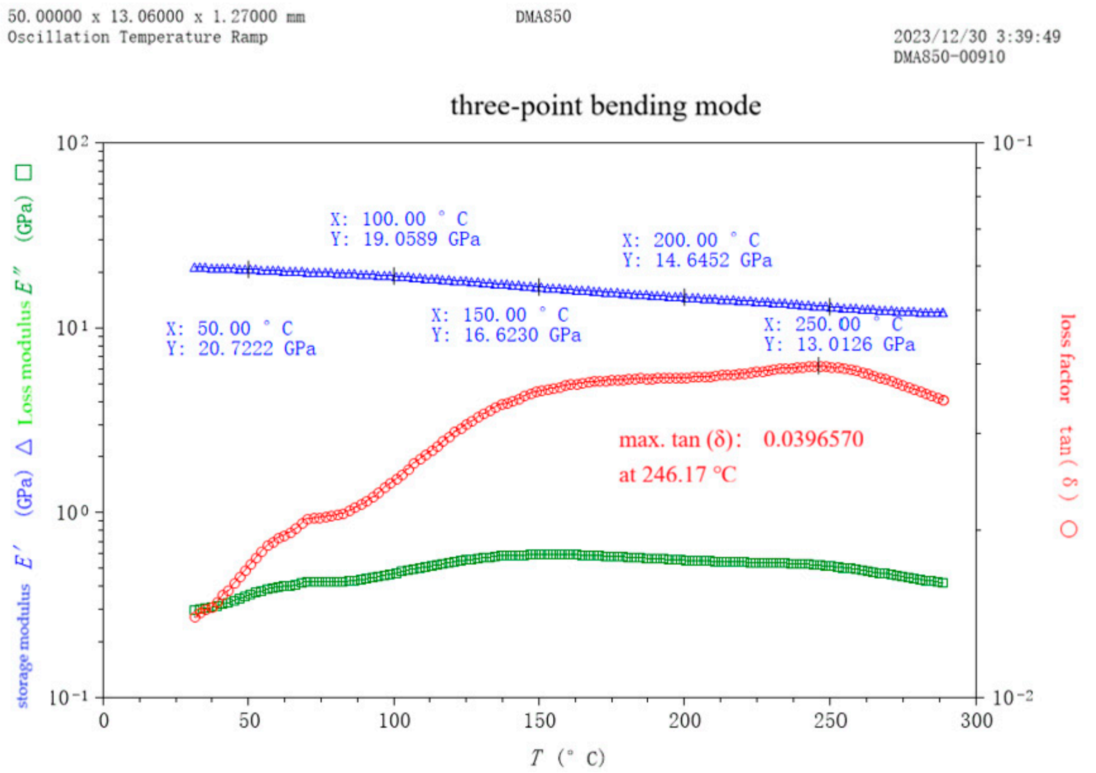

Figure S13. DMA curves of BP TVCH/OPE-2st CCLs measured by three-point bending mode on a TA Discovery DMA 850 under nitrogen flow (1 Hz, heating rate 3 °C/min, 30–300 °C).
